# Exhausted and trapped in isolation. Caring for a spouse with dementia during the Covid-19 pandemic

**DOI:** 10.1093/geroni/igab046.2932

**Published:** 2021-12-17

**Authors:** Lena Hammar, Marcus Johansson, Lena Dahlberg, Kevin J McKee, Martina Summer-Meranius

**Affiliations:** 1 Mälardalen University / Dalarna University, Västerås, Vastmanlands Lan, Sweden; 2 Dalarna University, Falun, Dalarnas Lan, Sweden; 3 Mälardalen University, Västerås, Vastmanlands Lan, Sweden

## Abstract

Even before the Covid-19 pandemic, spouse carers of persons with dementia (PwDs) found their care responsibilities overwhelming and had little time to focus on their own lives. To minimize the risk of being infected with Covid-19, older persons are recommended to self-isolate in their homes, while formal support such as respite care and day care centers are withdrawn. This study involved semi-structured interviews with 24 spouse carers of community-living PwDs, with the aim of describing their situation during the pandemic. The interviews were analyzed with qualitative content analysis. Results revealed that they commonly declined help because of the perceived risk of their spouse being infected with Covid-19 and thus also possibly causing their death. They described feelings of being trapped in their situation, as they experienced having no choice than take all responsibility for the care of their partner themselves, with cost of being unable to take necessary breaks. This was described as making an already strained situation almost unbearable, which led to conflicts with their partner. However, the spouses also described positive aspects due to strategic changes in health and social care provision to prevent the spread of the virus, such as greater staff continuity in home care services, and patient transportation service. These made the PwD less stressed and influenced their everyday life positively. It could be concluded that the extent burden during the Covid-19 pandemic calls for extensive development of tailored support to better tackle the rapid changes that can occur in a society.

